# Dietary Intake of Adults Who Participate in CrossFit^®^ Exercise Regimens

**DOI:** 10.3390/sports10030038

**Published:** 2022-03-05

**Authors:** Regis C. Pearson, Nathan T. Jenkins

**Affiliations:** Department of Kinesiology, University of Georgia, Athens, GA 30602, USA; regis.pearson@uga.edu

**Keywords:** diet, CrossFit^®^, high-intensity functional training, food frequency questionnaire

## Abstract

Background: The combination of high-intensity aerobic and high-load resistance training, as in CrossFit^®^, exerts similar or superior benefits to other exercise modalities. This study aimed to assess dietary habits and characterize the nutritional goals, exercise habits, and clinical health outcomes of individuals who participate in CrossFit^®^. Methods: Adults who are 19 y or older, with >6 mo of CrossFit^®^ participation, completed an electronic survey and the dietary health questionnaire III. In separate models, multiple stepwise linear regressions were performed to detect the associations between (i) dietary intake, (ii) exercise habits, (iii) clinical measures, and a priori selected predictors (sex, weight status, age, and exercise frequency) in each case. Odds ratios were detected between nutritional and fitness goals, clinical outcomes, and predictors. Results: In total, 449 respondents completed both questionnaires. Of these, 443 respondents were used for relative macronutrients assessment due to not reporting body weight. Dietary intake was associated with sex, weight status, age, exercise frequency, and nutritional goals. Nutritional and fitness goals and clinical outcomes were associated with sex, weight status, age, and exercise frequency. Conclusion: Nutritional goals are underlying factors that affect eating behaviors in non-competitive CrossFit^®^ participants. It is imperative to consider the sex, age, exercise habits, and nutritional goals of CrossFit^®^ participants when investigating and prescribing dietary outcomes.

## 1. Introduction

High-intensity functional training (HIFT) is rapidly growing in popularity as a viable exercise regimen. HIFT emphasizes functional, multi-joint movements that can be modified to any fitness level via exercises that utilize both aerobic and anaerobic metabolic systems to improve general physical fitness and performance [1]. Most studies that employ a HIFT methodology use a CrossFit^®^ exercise template. CrossFit^®^ is a strength and conditioning regimen that uses constantly varied, functional movements executed at high intensity [2] to improve an individual’s work capacity over various time domains and exercise modalities [3]. CrossFit^®^ utilizes monostructural, weightlifting, and gymnastics movements to enhance general physical skills (e.g., cardiovascular endurance, stamina, strength, flexibility, power, speed, coordination, agility, balance, and accuracy), the performance of athletic tasks, and competency of each metabolic system (e.g., the phosphagen, glycolytic, and oxidative systems) [4]. CrossFit^®^ provides a unique stimulus for physiological adaptation that has no equivalent in other forms of strength training, due to the blended variety of metabolic demands imposed [5]. Although CrossFit^®^ and other HIFT modalities have been growing in importance in the fitness industry for the last two decades, it is only recently that studies on the efficacy of HIFT have begun to emerge. A recent network meta-analysis of 45 studies concluded that the combination of high-intensity aerobic and high-load resistance training exerts benefits that are superior to other exercise modalities for decreasing body weight, body mass index (BMI), body fat percentage, and increasing lean body mass and cardiorespiratory fitness [6].

Adults engaging in general fitness programs are recommended to meet specific nutritional requirements, following a sports nutrition diet (i.e., 45–55% carbohydrate (CHO) (3–5 g·kg^−1^·d^−1^), 15–20% protein (PRO) (0.8–1.2 g·kg^−1^·d^−1^), and 25–35% fat (0.5–1.5 g·kg^−1^·d^−1^)) [7]. Higher PRO consumption (1.2–2.0 g·kg^−1^·d^−1^) has been suggested to positively affect body composition when coupled with exercise [8]. While high CHO intake is associated with greater exercise performance in prolonged and high-intensity intermittent single-modality exercise, it is still a matter of debate how necessary CHO intake is for participation in HIFT, specifically for adults trying to lose weight. Carbohydrate restriction for treating metabolic disease and weight loss has been proposed as the primary dietary treatment strategy [9].

There is currently limited interventional data supporting specific dietary strategies for CrossFit^®^ participants [10,11,12]. CrossFit^®^ suggests that daily intake should consist of 40% CHO, 30% PRO, and 30% fats, which is similar to the Zone diet [13,14]. Interestingly, these dietary recommendations do not match the CHO and energy requirements suggested by the International Society of Sports Nutrition [7]. We speculate that the lack of cohesive messaging about dietary recommendations for participants in CrossFit^®^ and HIFT has led to confusion about best practices and a general mismatching of dietary strategies for specific health and fitness goals in this population.

Therefore, to gain insight into the specific factors that influence the dietary intake of non-competitive CrossFit^®^ participants, the study’s purpose was to assess the dietary habits of individuals who have participated in CrossFit^®^ exercise for at least six months. Additionally, the investigators sought to characterize the nutritional goals, exercise habits, and clinical health outcomes among study participants. We hypothesized that non-competitive CrossFit^®^ participants’ dietary intake would be influenced by their sex, age, weight status, and exercise frequency. Additionally, we hypothesized that non-competitive CrossFit^®^ participants’ dietary intake would be correlated with their nutritional goals (i.e., lower energy intake will be positively associated with the intent to lose weight).

## 2. Materials and Methods

### 2.1. Ethical Approval

This study was approved by the University of Georgia Institutional Review Board (study no. 2964, approval date: 21 October 2020), with electronic informed consent being obtained before initiating any survey.

### 2.2. Study Design

This observational study was designed to reach the largest sample of participants and provide a large cross-sectional sample of CrossFit^®^ participants. For this purpose, we used a combination of an electronic intake survey tool using Qualtrics, LL.C (Provo, UT, USA) and the dietary health questionnaire (DHQ) III [15]. Adults aged 19 y or older, with more than six months of CrossFit^®^ participation, were asked to participate in this study. We used snowball sampling to distribute our survey among CrossFit^®^ community members using social media outlets, emails, and word of mouth [16]. Participants completed the intake survey, including listing their email address as primary contact for follow-up DHQ III participation. Data collection lasted from October 2020 until January 2021. Upon completing the DHQ III, participants were entered into a raffle for one of ten $50 gift cards to Amazon.com, Inc. (Seattle, WA, USA).

### 2.3. Intake Survey

We created an electronic intake survey to determine inclusion and collect demographic, nutritional goals, exercise habits, fitness goals, and clinical health data. Respondents could select nutritional goals such as losing fat mass, weight loss, supporting performance, weight maintenance, gaining muscle mass, and gaining weight. Respondents could also select cardiovascular endurance, overall wellbeing, and strength as fitness goals. The intake survey was also used to collect participants’ email addresses so that a unique link could be delivered to complete the food frequency questionnaire. The intake survey can be found as File S1: Intake Survey in the Supplementary Materials.

### 2.4. Food Frequency Questionnaire

The DHQ III is a self-administered web-based food frequency questionnaire developed by the National Cancer Institute that utilizes a nutrient and food group database from a compilation of national 24-h dietary recall data from the national health and nutrition examination surveys (NHANES 2007–2008, 2009–2010, 2011–2012, and 2013–2014), and the USDA’s food and nutrient database for dietary studies (FNDDS 2007–2008, 2009–2010, 2011–2012, and 2013–2014) [17]. The DHQ III consisted of reporting the past year’s dietary intake, with a portion size of 135 food and beverage line items and 26 dietary supplement questions.

### 2.5. Statistical Analysis

Means and SDs were calculated for different sex groups. Sex was categorized as male vs. female. Exercise frequency was calculated by summarizing the days per week of CrossFit^®^ class participation and of aerobic and strength exercise sessions other than CrossFit^®^. To assess dietary quality, the daily intake of each vitamin and mineral was divided by RDA or AI. An average of all vitamin and mineral adequacy percentages were divided by total energy intake, to estimate the quality of each respondent’s diet (e.g., micronutrient score = ((intake/RDA or AI) * 100)/kcals·d^−1^). The relative macronutrient level was calculated from absolute intake divided by the bodyweight of the respondent. Student’s *t*-tests, with an emphasis on gender (male vs. female), were conducted to assess the statistical significance of continuous variables. In separate models, multiple stepwise linear regressions were performed to detect the associations between: (i) diet and a priori selected predictors (e.g., sex, BMI, age, exercise frequency, and nutritional goals); (ii) exercise habits and a priori selected predictors (e.g., sex, BMI, age, and fitness goals); and (iii) clinical measures (e.g., heart rate and blood pressure) and a priori selected predictors (e.g., sex, BMI, age, and exercise frequency). If heteroscedasticity was detected, a weighted multiple linear regression was performed. Weighted multiple linear regressions were adjusted by the amount of prediction error associated with the dependent variable, to control for heteroscedasticity in the unweighted model. Binary stepwise regression, using the likelihood ratio, and logistic regression were performed to detect the ratios between nutritional and fitness goals, clinical outcomes (e.g., disease prevalence and medication), and a priori selected predictors (e.g., sex, BMI, age, and exercise frequency). Statistical significance was accepted at *p* ≤ 0.05; *p*-values are reported as 2-tailed unless otherwise noted. Data are reported as mean (SD) unless otherwise noted. All statistical analyses were performed with the SPSS statistics program, version 27.0 (IBM Corp., Armonk, NY, USA). In the following analysis, BMI is measured as kg·m^2^, age is measured in y, exercise frequency is measured in sessions·week^−1^, sex is coded as 0 = female, 1 = male, and nutritional goals are coded as 0 = no, 1 = yes.

## 3. Results

### 3.1. Respondents’ Characteristics

In total, 719 respondents completed the intake survey, with 450 respondents completing the DHQ III. Only data from those respondents who completed both the intake survey and DHQ III were retained for analysis. One respondent was excluded from the analysis due to abnormal dietary reporting. Of the remaining responses, 443 respondents were used for relative macronutrient assessment, due to not reporting body weight. The respondents’ physical characteristics, including the reported age, BMI, waist to hip ratio, resting heart rate, blood pressure, nutritional goal length, and exercise frequency, are displayed in Table 1.

### 3.2. Nutritional Evaluation

A summary of specific macronutrient intake can be found in Table 2. A summary of the multiple linear regression formulas, with dietary intake as the dependent variable, can be found in Table S1 in the Supplementary Materials. Correlations between dietary intake, exercise habits, resting heart rate, blood pressure, sex, BMI, age, exercise frequency, and nutritional and fitness goals are reported in the Supplementary Materials in Table S2: Correlations between dietary intake, exercise habits, resting heart rate, blood pressure, sex, BMI, age, exercise frequency, and nutritional and fitness goals.

#### 3.2.1. Energy Intake

A multiple linear regression was calculated to predict the absolute energy intake (kcals·d^−1^) based on sex, BMI, age, exercise frequency, and nutritional goals (F(3,439) = 28.100, *p* < 0.001, R^2^ = 0.161). The respondents’ predicted absolute energy intake is equal to 2261.87 + 540.70 (sex) − 14.95 (age) + 411.19 (goal: weight gain), with sex, age, and a nutritional goal of weight gain being significant predictors of absolute energy intake (*p* < 0.001, *p* < 0.001, and *p* = 0.022, respectively). More details can be found in the Supplementary Materials in Table S3: Results of multiple linear regression analysis with absolute energy and carbohydrate, fat, and alcohol percentages of total energy as the dependent variables.

A weighted multiple linear regression was calculated to predict the relative energy intake (kcals·kg^−1^·d^−1^) based on sex, BMI, age, exercise frequency, and nutritional goals (F(6,436) = 15.211, *p* < 0.001, R^2^ = 0.173). The respondents’ predicted relative energy intake is equal to 46.21 − 0.64 (BMI) − 0.13 (age) + 0.34 (exercise frequency) − 1.98 (goal: lose fat mass) + 6.50 (goal: weight gain) − 2.56 (goal: weight loss), with BMI, age, exercise frequency, and a nutritional goal of lose fat mass, weight gain and weight loss being significant predictors of relative energy intake (*p* < 0.001, *p* < 0.001, *p* = 0.031, *p* = 0.020, *p* = 0.024, *p* = 0.030, respectively). More details can be found in the Supplementary Materials in Table S5: Results of weighted multiple linear regression analysis with relative macronutrient variables as the dependent variables.

#### 3.2.2. CHO and Fiber Intake

Multiple linear regression was calculated to predict the percentage of energy drawn from CHO (% energy) based on sex, BMI, age, exercise frequency, and nutritional goals (F(4,438) = 9.148, *p* < 0.001, R^2^ = 0.077). The respondents’ predicted percentage of energy from CHO is equal to 46.49 − 2.56 (sex) − 0.15 (age) + 0.32 (exercise frequency) + 4.47 (goal: weight gain), with sex, age, exercise frequency, and a nutritional goal of weight gain being significant predictors of the percentage of energy from CHO (*p* < 0.001, *p* = 0.006, *p* = 0.038, and *p* = 0.046, respectively). More details can be found in the Supplementary Materials in Table S3: Results of multiple linear regression analysis with absolute energy and carbohydrate, fat, and alcohol percentages of total energy as the dependent variables.

Multiple linear regression was calculated to predict the absolute CHO intake (g·d^−1^) based on sex, BMI, age, exercise frequency, and nutritional goals (F(5,437) = 16.169, *p* < 0.001, R^2^ = 0.156). The respondents’ predicted absolute CHO intake is equal to 242.92 + 45.33 (sex) − 2.19 (age) + 3.98 (exercise frequency) − 22.54 (goal: weight loss) + 66.98 (goal: weight gain), with sex, age, exercise frequency, and a nutritional goal of weight loss and weight gain being significant predictors of absolute CHO intake (*p* < 0.001, *p* < 0.001, *p* = 0.015, *p* = 0.039 and *p* = 0.005, respectively). More details can be found in the Supplementary Materials in Table S4: Results of multiple linear regression analysis with absolute macronutrient and micronutrient score variables as the dependent variables.

A weighted multiple linear regression was calculated to predict the relative CHO intake (g·kg^−1^·d^−1^) based on sex, BMI, age, exercise frequency, and nutritional goals (F(5,437) = 22.860, *p* < 0.001, R^2^ = 0.207). The respondents’ predicted relative CHO intake is equal to 4.61 − 0.05 (BMI) − 0.02 (age) + 0.05 (exercise frequency) − 0.41 (goal: weight loss) + 1.18 (goal: weight gain), with BMI, age, exercise frequency, and a nutritional goal of weight loss and weight gain being significant predictors of relative CHO intake (*p* < 0.001, *p* < 0.001, *p* = 0.018, *p* = 0.002 and *p* < 0.001, respectively). More details can be found in the Supplementary Materials in Table S5: Results of weighted multiple linear regression analysis with relative macronutrient variables as the dependent variables.

Multiple linear regression was calculated to predict absolute dietary fiber intake (g·d^−1^) based on sex, BMI, age, exercise frequency, and nutritional goals (F(5,437) = 13.346, *p* < 0.001, R^2^ = 0.1320. The respondents’ predicted absolute dietary fiber intake is equal to 24.76 + 5.23 (sex) − 0.21 (age) + 0.63 (exercise frequency) − 3.23 (goal: lose fat mass) + 3.09 (goal: support performance), with sex, age, exercise frequency, and a nutritional goal as lose fat mass and support performance being significant predictors of reported absolute dietary fiber (*p* < 0.001, *p* < 0.001, *p* = 0.002, *p* = 0.005 and *p* = 0.013, respectively). More details can be found in the Supplementary Materials in Table S6: Results of multiple linear regression analysis with dietary fiber, SFA, MUFA, PUFA, and CHOL as the dependent variables.

#### 3.2.3. PRO Intake

Multiple linear regression was calculated to predict the percentage of energy from PRO (% energy) based on sex, BMI, age, exercise frequency, and nutritional goals. No significant regression equation was found (*p* > 0.05).

Multiple linear regression was calculated to predict the absolute PRO intake (g·d^−1^) based on sex, BMI, age, exercise frequency, and nutritional goals (F(3,439) = 24.057, *p* < 0.001, R^2^ = 0.135). The respondents’ predicted absolute PRO intake is equal to 109.34 + 34.04 (sex) − 0.77 (age) + 1.82 (exercise frequency), with sex, age, and exercise frequency being significant predictors of absolute PRO intake (*p* < 0.001, *p* < 0.001, and *p* = 0.020, respectively). More details can be found in the Supplementary Materials in Table S4: Results of multiple linear regression analysis with absolute macronutrient and micronutrient score variables as the dependent variables.

A weighted multiple linear regression was calculated to predict the relative PRO intake (g·kg^−1^·d^−1^) based on sex, BMI, age, exercise frequency, and nutritional goals (F(4,438) = 13.810, *p* < 0.001, R^2^ = 0.112). The respondents’ predicted relative PRO intake is equal to 4.61 + 0.20 (sex) − 0.05 (BMI) − 0.01 (age) + 0.02 (exercise frequency), with sex, BMI, age, and exercise frequency being significant predictors of relative PRO intake (*p* = 0.002, *p* < 0.001, *p* = 0.002, and *p* = 0.032, respectively). More details can be found in the Supplementary Materials in Table S5: Results of weighted multiple linear regression analysis with relative macronutrient variables as the dependent variables.

#### 3.2.4. Fat Intake

Multiple linear regression was calculated to predict the percentage of energy from fat (% energy) based on sex, BMI, age, exercise frequency, and nutritional goals (F(3,439) = 10.053, *p* < 0.001, R^2^ = 0.064). Respondents’ predicted percentage of energy from fat is equal to 30.67 − 2.11 (sex) − 0.12 (age) − 3.52 (goal: weight gain), with sex, age, and a nutritional goal of weight gain being significant predictors of the percentage of energy from fat (*p* = 0.005, *p* < 0.001, and *p* = 0.049, respectively). More details can be found in the Supplementary Materials in Table S3: Results of multiple linear regression analysis with absolute energy, and carbohydrate, fat, and alcohol percentages of total energy as the dependent variables.

A weighted multiple linear regression was calculated to predict absolute fat intake (g·d^−1^) based on sex, BMI, age, exercise frequency, and nutritional goals (F(3,439) = 19.728, *p* < 0.001, R^2^ = 0.119). The respondents’ predicted absolute fat intake is equal to 75.31 + 23.74 (sex) − 0.34 (age) + 6.26 (goal: support performance), with sex, age, and a nutritional goal of support performance being significant predictors of absolute fat intake (*p* < 0.001, *p* = 0.003, and *p* = 0.048, respectively). More details can be found in the Supplementary Materials in Table S4: Results of multiple linear regression analysis with absolute macronutrient and micronutrient score variables as the dependent variables.

A weighted multiple linear regression was calculated to predict relative fat intake (g·kg^−1^·d^−1^) based on sex, BMI, age, exercise frequency, and nutritional goals (F(3,439) = 29.729, *p* < 0.001; R^2^ = 0.169). The respondents’ predicted relative fat intake is equal to 1.93 + 0.14 (sex) − 0.03 (BMI) − 0.004 (age), with sex, BMI, and age being significant predictors of relative fat intake (*p* < 0.001, *p* < 0.001, and *p* = 0.011, respectively). More details can be found in the Supplementary Materials in Table S5: Results of weighted multiple linear regression analysis with relative macronutrient variables as the dependent variables.

#### 3.2.5. Fatty Acid Intake

A weighted multiple linear regression was calculated to predict absolute saturated fatty acid intake (SFA; g·d^−1^) based on sex, BMI, age, exercise frequency, and nutritional goals (F(2,440) = 20.256, *p* < 0.001, R^2^ = 0.084). The respondents’ predicted absolute SFA intake is equal to 23.61 + 6.57 (sex) − 0.10 (age), with sex and age being significant predictors of absolute SFA intake (*p* < 0.001 and *p* = 0.009, respectively). More details can be found in the Supplementary Materials in Table S6: Results of multiple linear regression analysis with dietary fiber, SFA, MUFA, PUFA, and CHOL as the dependent variables.

A weighted multiple linear regression was calculated to predict absolute monounsaturated fatty acid intake (MUFA; g·d^−1^) based on sex, BMI, age, exercise frequency, and nutritional goals (F(3,439) = 19.643, *p* < 0.001, R^2^ = 0.118). The respondents’ predicted absolute MUFA intake is equal to 27.42 + 10.17 (sex) − 0.10 (age) + 2.73 (goal: support performance), with sex, age, and a nutritional goal of support performance being significant predictors of absolute MUFA intake (*p* < 0.001, *p* = 0.039, and *p* = 0.039, respectively), Table S6 Results of multiple linear regression analysis with dietary fiber, SFA, MUFA, PUFA, and CHOL as the dependent variables.

Multiple linear regression was calculated to predict absolute polyunsaturated fatty acid intake (PUFA; g·d^−1^) based on sex, BMI, age, exercise frequency, and nutritional goals (F(4,438) = 17.247, *p* < 0.001, R^2^ = 0.136). The respondents’ predicted absolute PUFA intake is equal to 16.82 + 4.89 (sex) − 0.10 (age) + 0.41 (exercise frequency) − 1.49 (goal: lose fat mass), with sex, age, exercise frequency, and a nutritional goal of lose fat mass being significant predictors of absolute PUFA intake (*p* < 0.001, *p* = 0.001, *p* = 0.001, and *p* = 0.042, respectively), Table S6 Results of multiple linear regression analysis with dietary fiber, SFA, MUFA, PUFA, and CHOL as the dependent variables.

A weighted multiple linear regression was calculated to predict absolute cholesterol intake (CHOL; mg·d^−1^) based on sex, BMI, age, exercise frequency, and nutritional goals (F(2,440) = 17.224, *p* < 0.001, R^2^ = 0.073). The respondents’ predicted absolute CHOL intake is equal to 273.24 + 135.05 (sex) + 51.05 (goal: support performance), with sex and a nutritional goal of support performance being significant predictors of absolute CHOL intake (*p* < 0.001 and *p* = 0.026, respectively). More details can be found in the Supplementary Materials in Table S6: Results of multiple linear regression analysis with dietary fiber, SFA, MUFA, PUFA, and CHOL as the dependent variables.

#### 3.2.6. Alcohol Intake

A weighted multiple linear regression was calculated to predict the percentage of energy from alcohol (% energy) based on sex, BMI, age, exercise frequency, and nutritional goals (F(2,440) = 43.477, *p* < 0.001, R^2^ = 0.165). The respondents’ predicted percentage of energy from alcohol is equal to 1.31 + 0.05 (age) − 0.11 (exercise frequency), with age and exercise frequency being significant predictors of the percentage of energy from alcohol (*p* < 0.001). More details can be found in the Supplementary Materials in Table S3: Results of multiple linear regression analysis with absolute energy and carbohydrate, fat, and alcohol percentages of total energy as the dependent variables.

A weighted multiple linear regression was calculated to predict absolute alcohol intake (g·d^−1^) based on sex, BMI, age, exercise frequency, and nutritional goals (F(2,440) = 15.012, *p* < 0.001, R^2^ = 0.064). The respondents’ predicted absolute alcohol intake is equal to 3.68 + 0.11 (age) − 0.31 (exercise frequency), with age and exercise frequency being significant predictors of absolute alcohol intake (*p* < 0.001). More details can be found in the Supplementary Materials in Table S4: Results of multiple linear regression analysis with absolute macronutrient and micronutrient score variables as the dependent variables.

A weighted multiple linear regression was calculated to predict the relative alcohol intake (g·kg^−1^·d^−1^) based on sex, BMI, age, exercise frequency, and nutritional goals (F(2,440) = 14.437, *p* < 0.001, R^2^ = 0.062). The respondents’ predicted relative alcohol intake is equal to 10.06 + 0.001 (age) − 0.004 (exercise frequency), with age and exercise frequency being significant predictors of relative alcohol intake (*p* < 0.001 and *p* = 0.002, respectively). More details can be found in the Supplementary Materials in Table S5: Results of weighted multiple linear regression analysis with relative macronutrient variables as the dependent variables.

#### 3.2.7. Vitamin and Mineral Intake

A multiple linear regression was calculated to predict the micronutrient score based on sex, BMI, age, exercise frequency, and nutritional goals (F(4,438) = 8.509, *p* < 0.001, R^2^ = 0.072). The respondents’ predicted micronutrient score is equal to 0.101 − 0.01 (sex) − 0.001 (BMI) + 0.001 (exercise frequency) + 0.01 (goal: weight loss), with sex, BMI, exercise frequency, and a nutritional goal of weight loss being significant predictors of micronutrient score (*p* = 0.004, *p* = 0.003, *p* = 0.011, and *p* = 0.013, respectively). More details can be found in the Supplementary Materials in Table S4: Results of multiple linear regression analysis with absolute macronutrient and micronutrient score variables as the dependent variables. Specific vitamin and mineral intake are reported in the Supplementary Materials in Table S7: Vitamin and mineral intake used to calculate micronutrient scores.

#### 3.2.8. Estimation of Nutritional Under-Reporting of Dietary Intake

The historical disparities of underreporting nutritional intake are greatest among differing weight statuses, specifically individuals with obesity [17]. We categorized respondents with a BMI of <27.0 as normal weight status and respondents with a BMI of ≥27.0 as overweight/obese (OW/OB). We estimated the resting energy expenditure (REE) from respondents’ demographics [18] and multiplied by 1.3 for physical activity level, then compared our estimated energy need to the reported energy intake. Among all respondents, the estimated REE of those with normal weight status and OW/OB respondents was higher compared to the reported energy intake (kcals·d^−1^, *p* = 0.003). While OW/OB respondents underreported by 336.52 (1191.45) kcals·d^−1^, respondents with normal weight status underreported only 192.49 (739.95) kcals·d^−1^. The magnitude of estimated underreporting was not statistically significantly different between normal-weight and OW/OB respondents *(p* = 0.130).

### 3.3. Nutritional and Fitness Goals, Exercise Habits, and Clinical Outcomes

A summary of the regression formulae, with exercise habits and clinical outcomes as the dependent variables, can be found in Table 3.

#### 3.3.1. Nutritional and Fitness Goals

Sex, BMI, age, and exercise frequency significantly affected the likelihood of selecting specific nutritional goals (Table 4). Additionally, sex, age, and exercise frequency significantly affected the likelihood of selecting specific fitness goals (Table 4).

#### 3.3.2. Exercise Habits

Multiple linear regression was calculated to predict total exercise sessions·wk-1 based on sex, BMI, age, and fitness goals (F(2,440) = 11.795, *p* < 0.001, R^2^ = 0.051). The respondents’ predicted total exercise sessions·wk-1 are equal to 7.59 + 1.10 (goal: cardiovascular endurance) − 1.05 (goal: overall wellbeing), with a fitness goal of cardiovascular endurance and overall wellbeing as significant predictors of total exercise sessions·week^−1^ (*p* < 0.001, and *p* = 0.001, respectively). More details can be found in the Supplementary Materials in Table S8: Results of multiple linear regression analysis with total exercise sessions and additional aerobic and strength exercise sessions as the dependent variables.

Multiple linear regression was calculated to predict CrossFit^®^ class frequency based on sex, BMI, age, and fitness goals. No significant regression equation was found (*p* > 0.05).

A multiple linear regression was calculated to predict the additional aerobic exercise sessions·week^−1^ other than CrossFit^®^ sessions, based on sex, BMI, age, and fitness goals (F(2,440) = 9.287, *p* < 0.001, R^2^ = 0.041). The respondents’ predicted additional aerobic exercise sessions·week^−1^ other than CrossFit^®^ sessions are equal to 1.53 + 0.63 (goal: cardiovascular endurance) − 0.43 (goal: overall wellbeing), with a fitness goal of cardiovascular endurance and overall wellbeing as significant predictors of additional aerobic exercise sessions·week^−1^ other than CrossFit^®^ sessions (*p* < 0.001, and *p* = 0.024, respectively). More details can be found in the Supplementary Materials in Table S8: Results of multiple linear regression analysis with total exercise sessions and additional aerobic and strength exercise sessions as the dependent variables.

Multiple linear regression was calculated to predict additional strength exercise sessions·week^−1^ other than CrossFit^®^ sessions based on sex, BMI, age, and fitness goals (F(2,440) = 12.619, *p* < 0.001, R^2^ = 0.054). The respondents’ predicted additional strength exercise sessions·week^−1^ other than CrossFit^®^ sessions are equal to 1.73 + 0.39 (goal: cardiovascular endurance) − 0.81 (goal: overall wellbeing), with a fitness goal of cardiovascular endurance and overall wellbeing being significant predictors of additional strength exercise sessions·week^−1^ other than CrossFit^®^ sessions (*p* = 0.013, and *p* < 0.001, respectively). More details can be found in the Supplementary Materials in Table S8: Results of multiple linear regression analysis with total exercise sessions and additional aerobic and strength exercise sessions as the dependent variables.

#### 3.3.3. Disease and Prescriptions

Multiple linear regression was calculated to predict the resting heart rate (bpm), based on sex, BMI, age, and exercise frequency (F(3,392) = 16.488, *p* < 0.001, R^2^ = 0.112). The respondents’ predicted resting heart rate is equal to 51.21 − 4.78 (sex) + 0.40 (BMI) − 0.52 (exercise frequency), with sex, BMI, and exercise frequency being significant predictors of reported resting heart rate (*p* < 0.001). More details can be found in the Supplementary Materials in Table S9: Results of multiple linear regression analysis with heart rate and blood pressure as the dependent variables.

Multiple linear regression was calculated to predict systolic blood pressure (mmHg) based on sex, BMI, age, and exercise frequency (F(2,254) = 25.633, *p* < 0.001, R^2^ = 0.168). The respondents’ predicted systolic blood pressure is equal to 99.15 + 6.99 (sex) + 0.47 (BMI), with sex and BMI being significant predictors of reported systolic blood pressure (*p* < 0.001 and *p* = 0.001, respectively). More details can be found in the Supplementary Materials Table S9: Results of multiple linear regression analysis with heart rate and blood pressure as the dependent variables.

Multiple linear regression was calculated to predict diastolic blood pressure (mmHg), based on sex, BMI, age, and exercise frequency (F(2,254) = 12.321, *p* < 0.001, R^2^ = 0.088). The respondents’ predicted diastolic blood pressure is equal to 59.39 + 2.75 (sex) + 0.39 (BMI), with sex and BMI being significant predictors of reported diastolic blood pressure (*p* = 0.009 and *p* < 0.001, respectively). More details can be found in the Supplementary Materials in Table S9: Results of multiple linear regression analysis with heart rate and blood pressure as the dependent variables.

Sex, BMI, and age influenced the likelihood of reporting a diagnosis of mental, cardiovascular, metabolic, or skeletomuscular disease, taking prescription medications (including birth control), change in diagnosis or medication after participating in CrossFit^®^, or change in disease symptoms after participating in CrossFit^®^ (Table 5).

An additional analysis was performed for prescription medication usage, excluding birth control. Males were less likely to report taking prescription medications (excluding birth control) (OR: 0.349 (95% CI: 0.212, 0.574)) and older respondents were more likely to report taking prescription medications (excluding birth control) (OR: 1.040 (95% CI: 1.021, 1.060)). While presenting similar trends, prescription usage excluding birth control is not reported due to the model’s accounting for less variance (e.g., excluding birth control, Nagelkerke R^2^ = 0.098 and including birth control, Nagelkerke R^2^ = 0.123).

## 4. Discussion

In the current study, we assessed the dietary habits of individuals who have participated in a CrossFit^®^ regimen for at least six months. Additionally, we sought to characterize study respondents’ nutritional goals, exercise habits, and clinical health outcomes. The major finding of this study is that dietary intake was associated with sex, weight status, age, exercise frequency, and nutritional goals. Nutritional and fitness goals and clinical outcomes (e.g., disease diagnosis) were associated with sex, weight status, age, and exercise frequency. Exercise habits were associated with the fitness goals of non-competitive CrossFit^®^ participants. Overall, these data suggest that genetic and lifestyle factors are related to dietary intake, the incidence of disease, and clinical outcomes.

We hypothesized that non-competitive CrossFit^®^ participants’ dietary intake would be influenced by sex, age, weight status, exercise frequency, and nutritional goals. Our data generally supported this hypothesis, with males reporting higher total energy and amounts of macronutrients, age being associated with an overall decreased intake, and exercise frequency being associated with increased energy, CHO, and PRO intake. The sex differences reported here are consistent with established reports of dietary intake of trained CrossFit^®^ participants, except for the percentage of total calories from fat [10]. The dietary intake of non-competitive CrossFit^®^ participants follows similar patterns to other populations of physically active and sedentary adults.

Contrary to our hypothesis, some dietary outcomes (e.g., energy, macronutrient, and micronutrient intake) were inversely influenced by weight status. We speculate two possibilities for these results: (1) underreporting of dietary intake by individuals with higher body mass index, and (2) the use of the body mass index may misrepresent weight status in CrossFit^®^ participants. Individuals with increased weight status have been reported to underreport their dietary intake by ~200–700 kcals·d^−1,^ with obese individuals underreporting to a greater extent [18]. We chose to estimate the resting energy expenditure from respondents’ demographics and compared this against the reported energy intake to investigate this plausibility. While we did find underreporting in both normal and overweight/obese respondents, these values did not statistically differ; thus, this reduced the likelihood of underreporting driving the weight status’s influence on dietary intake. The use of the body mass index may also misrepresent weight status in trained individuals [19]. We speculate that most overweight/obese respondents, as classified according to BMI, have a healthy body fat percentage. Future investigations should employ assessments of body composition to accurately classify the weight status in CrossFit^®^ participants.

Interestingly, age was positively associated with reported alcohol intake. Alcohol intake has been reported to be inversely associated with age [20], although it is suggested that older adults over 60 years of age have a high prevalence of binge drinking [21]. We speculate that the positive association noted in the current study is partially driven by the social environment fostered through CrossFit^®^. Additionally, exercise frequency was negatively associated with reported alcohol intake. We believe that the competitive nature of a CrossFit^®^ group atmosphere would drive participants to be concerned about performance and recovery strategies. The decrease in reported alcohol intake with increasing exercise frequency could be due to the adverse effects of alcohol on exercise performance and recovery [22,23]. Additional investigation is warranted into the underlying reasons for alcohol consumption in non-competitive CrossFit^®^ participants.

There are discrepancies between the CrossFit^®^ recommended dietary habits and other sports nutrition guidance, possibly leading to contrary influences on participants’ dietary intake. Although our current study follows similar dietary relationships and the influence of sex, age, and exercise frequency in exercising individuals [24,25,26], this discrepancy could impact the dietary reporting of CrossFit^®^ participants. When tested using traditional sports nutrition recommendations, CrossFit^®^ trainers answer about 65% of sports nutrition knowledge questions correctly, with most knowledgeable areas being energy needs/recovery and micronutrients, and they are least knowledgeable about hydration and macronutrients [14]. This is perhaps not surprising, since current recommendations from CrossFit^®^ oppose other sports nutrition guidelines [7,13]. Influences on an individual’s dietary choices are an important factor when evaluating if an individual is adhering to an appropriate diet. Emphasis should be put on education regarding the dietary needs of physically active and competitive athletes alike.

Individuals who report endorsing exercise as an essential goal have higher facilitation ratings for nutritional goals, suggesting that exercising and nutritional goals are linked as priorities [27]. In the current study, we hypothesized that non-competitive CrossFit^®^ participants’ dietary intake would be correlated with their nutritional goals (i.e., lower energy intake will be positively associated with the intent to lose weight). The nutritional goals of losing fat mass, losing weight, and gaining weight had the most influence on reported dietary intake, specifically regarding energy and CHO intake. The dietary intake of exercising individuals, specifically CrossFit^®^ participants, is influenced by nutritional goals directed to changing weight status or composition instead of goals that support performance and weight maintenance. Assessing nutritional goals is critical for understanding the dietary patterns of CrossFit^®^ participants.

High-intensity functional training programs, like CrossFit^®^, have shown a decrease in body fat and improved cardiorespiratory fitness and strength [28,29,30,31], along with higher enjoyment levels than traditional resistance training [32,33]. In the current study, the fitness goals of cardiovascular endurance and overall well-being influenced exercise characteristics. Surprisingly, respondents that selected overall well-being as a fitness goal reported lower amounts of total exercise, primarily from aerobic and strength exercise other than a CrossFit^®^ class. We speculate that this is due to the inclusive benefits of HIFT exercise programs (i.e., additional exercise may not feel necessary to achieve goals).

A high resting heart rate and high blood pressure are associated with a higher risk of cardiovascular morbidity and mortality [34]. In the current study, males had an association with lower self-reported resting heart rates and higher blood pressure. These findings support the literature in large populations regarding resting heart rate [35] and blood pressure [36], possibly due to the role of estrogen in the renin-angiotensin system [37]. In agreement with the previous literature on normal-population Americans [38], non-competitive CrossFit^®^ participants with increased weight status were associated with having a higher resting heart rate and higher blood pressure. Lastly, exercise participation decreases the resting heart rate [39]; our current work confirms this finding in non-competitive CrossFit^®^ participants. It appears that chronic participation in CrossFit^®^ has positive effects on resting heart rate and, thus, on cardiovascular disease risk.

To our knowledge, this is the first study to assess nutritional goals in non-competitive CrossFit^®^ participants. The nutritional goals we selected were theoretically linked to exercise performance, and the interaction between the two should not be separated. We found that sex is associated with the nutritional goals of weight or fat loss and weight or muscle gain. Specifically, males were less likely to select losing fat mass and weight loss and are more likely to select gaining muscle mass and gaining weight as their nutritional goals. These findings are consistent with previous research on exercising behavior, where females were reported to exercise for weight loss more often than males, and males were oriented on gaining muscle mass/weight [40,41,42]. Additionally, increased weight status was associated with nutritional goal selection. Specifically, individuals with higher BMI were more likely to select weight or fat loss and less likely to select gaining muscle mass and gaining weight as nutritional goals. These data support the idea that non-competitive CrossFit^®^ participants set nutritional goals to influence their weight status.

Muscle atrophy contributes to disability in older adults [43] and is accompanied by a progressive increase in fat mass, associated with an increased incidence of insulin resistance [44,45]. Interestingly, increased age was associated with a decreased likelihood of selecting the goals of gaining muscle mass or weight. Due to the negative aspects of muscle atrophy in older adults, compounded by their increased risk of inadequate dietary intake [46], it is paramount to further educate older adults on how dietary practices can influence their quality of life. Additionally, further investigation into additional factors for older adults’ nutritional goal choices should be investigated. Evaluating nutritional goals before and during a dietary intervention will help to tailor the diet to the needs of the individuals and further promote the long-term maintenance of a healthy lifestyle.

To our knowledge, this study is the first to evaluate the fitness goals of specific energy systems (e.g., cardiovascular endurance, strength, flexibility, and overall wellbeing) in an exercising population. Motivational goals can impact attitude, behavioral control, and expectations regarding exercise programs. In the current study, males were more likely to select cardiovascular endurance and flexibility as fitness goals. This is counter to our hypothesis since males have been reported to be oriented to gaining muscle mass/weight [40,41,42]. CrossFit^®^ promotes overall fitness in all energy domains. We speculate that these differences are due to males already perceiving themselves as having enough strength and realizing the need for greater cardiovascular endurance to perform better in CrossFit^®^.

Older adults differ in their behavior, beliefs, and motivational states toward structured exercise [47]. Interestingly, respondents of increased age were less likely to select cardiovascular endurance and strength as fitness goals but were more likely to select overall wellbeing as a fitness goal. We hypothesized that the goals of cardiovascular endurance, strength, and flexibility would contribute to overall wellbeing but this may not be the case with increasing age. One possibility of this discrepancy is the lack of education about how these components of fitness influence an individual’s overall wellbeing. Another possibility is that respondents in the current study do not perceive the selected components as essential to their overall wellbeing and may consider other components of wellbeing to be more critical, such as the intellectual or emotional aspects.

Interestingly, respondents with higher exercise frequency were less likely to select overall wellbeing as a fitness goal but were more likely to select cardiovascular endurance and flexibility as fitness goals. We believe that individuals participating in more exercise are more concerned with their performance; thus, they value individual components of fitness more than their overall wellbeing. These results speak to the importance of goal establishment before the evaluation of any dietary or fitness habits.

Epidemiological data have consistently reported higher rates of mood and mental disorders among females than males [48,49]. This finding is supported in the present study, where males were less likely to report having a mental disorder than females. Additionally, males were less likely to report taking prescription medication/s and having a change in the symptoms of disease after participating in CrossFit^®^. We speculate that these results are influenced by the incidence of mental disorders reported herein.

The shift in weight distribution toward obesity in the general population has led to an interest in the effects of weight status on physical health. There is a strong positive correlation between higher weight status and mental disorders, cardiovascular disease, and metabolic disease [50,51,52]. These data are supported in the present study of non-competitive CrossFit^®^ participants. Additionally, we found that individuals with a higher BMI were more likely to report changes in the symptoms of diseases and even changes in diagnoses after participating in CrossFit^®^. Further investigation is warranted to examine the underlying reasons for symptom and disease changes in participants of CrossFit^®^ with higher weight status.

Age is an independent risk factor for cardiovascular disease in adults [53]; this is supported in non-competitive CrossFit^®^ participants. Interestingly, respondents of increased age were associated with more likelihood of reporting taking prescription medications, changes in the symptoms of diseases, and changes in diagnoses after participating in CrossFit^®^. We speculate that older adults participating in CrossFit^®^, while reporting higher cardiovascular disease, benefit from participation by its positively affecting disease symptoms.

Limitations of this study do exist. The present cohort was partially a convenience sample of only CrossFit^®^ members, with no control group. This absence of data prevented any comparisons to a sedentary or other exercise modality population. These findings could be insightful to examine differences between physical activity and different exercise modalities. The data herein may be biased due to the respondents needing an internet connection to participate in the study. It is presumed that about half of CrossFit^®^ participants are female. In our current study, 64.6% of respondents identified as female; this may lead to a sex bias within our data. In addition, we did not assess the gender orientation of the sample. Gender identity or sexual orientation influences dietary habits [54] and may impact nutrition goals; therefore, gender identity should be considered in future studies. The current study was performed during the COVID-19 pandemic, possibly influencing dietary habits and the exercise frequency of respondents. We did not select a priori years of experience to predict dietary habits. Future investigations should consider years of experience in the evaluation of dietary habits in an exercising population. All demographic data were generated from self-reported metrics; this may lead to a response bias. To try to combat a possible response bias, we included descriptions of metrics that are not commonly taken by an individual, i.e., waist and hip circumferences (see Supplementary Material File S1: Intake Survey). Additionally, respondents were not trained in the assessment of serving sizes prior to completing the DHQ III. This could lead to inaccurate reporting of dietary intake, and future research should allow for educational opportunities for participants prior to completing a food frequency questionnaire that includes serving sizes. Lastly, the findings in the current study are correlative and should not be interpreted as a cause-effect relationship.

## 5. Conclusions

Assessing the goals of individuals when examining dietary outcomes is paramount. The purpose of the current study was to gain insight into non-competitive CrossFit^®^ participants’ dietary habits by assessing individuals who have participated in CrossFit^®^ exercise for at least six months. Additionally, investigators sought to characterize respondents’ nutritional goals, exercise habits, and clinical health outcomes. The major finding of this study is that dietary intake was associated with sex, weight status, age, exercise frequency, and nutritional goals. Overall, current data suggest that genetic and lifestyle factors are related to dietary intake, the incidence of disease, and clinical outcomes. Additional investigation is needed into how dietary habits and goals differ between CrossFit^®^ participants and other exercise programs. Nutritional goals are underlying factors that affect eating behaviors in non-competitive CrossFit^®^ participants.

## Figures and Tables

**Table 1:** Respondents’ characteristics.

	*n*	All	*n*	Female	*n*	Male	*p*-Value
Age (y)	449	36.55 (11.38)	290	35.37 (10.91)	159	38.69 (11.92)	*p* = 0.003
Body mass index (kg·m^2^)	443	25.56 (4.24)	286	24.95 (4.23)	157	26.67 (4.03)	*p* < 0.001
Waist-to-hip ratio	247	0.83 (0.08)	182	0.81 (0.07)	65	0.89 (0.06)	*p* < 0.001
Resting heart rate (bpm)	401	55.93 (8.98)	258	57.32 (8.83)	143	53.42 (8.72)	*p* < 0.001
SBP (mmHg)	260	113.84 (10.66)	164	110.88 (9.74)	96	118.90 (10.30)	*p* < 0.001
DBP (mmHg)		70.52 (8.13)		69.22 (8.08)		72.75 (0.79)	*p* < 0.001
CrossFit^®^ participation (y)	446	4.92 (3.08)	287	4.71 (3.08)	159	5.30 (3.04)	*p* > 0.05
Nutritional goal length (y)	422	2.55 (3.58)	270	2.33 (2.79)	152	2.96 (4.64)	*p* > 0.05
Total exercise sessions·week^−1^	449	7.53 (2.80)	290	7.46 (2.70)	159	7.67 (2.99)	*p* = 0.052
CrossFit^®^ sessions·week^−1^		4.54 (1.28)		4.45 (1.23)		4.65 (1.36)	*p* > 0.05
Additional strength sessions·week^−1^		1.36 (1.54)		1.32 (1.52)		1.43 (1.58)	*p* > 0.05
Additional aerobic sessions·week^−1^		1.63 (1.61)		1.65 (1.58)		1.60 (1.67)	*P* > 0.05

Note: y, years; bpm, beats·min^−1^; SBP, systolic blood pressure; DBP, diastolic blood pressure; mmHg, millimeters of mercury.

**Table 2:** Specific macronutrient intake reported.

	*n*	All	*n*	Female	*n*	Male	ANOVA
Energy (kcals·d^−1^)	449	1922.24 (790.16)	290	1739.42 (624.99)	159	2255.68 (939.47)	*p* < 0.001
CHO (% energy)		42.63 (9.31)		43.57 (8.98)		40.92 (9.67)	*p* = 0.004
CHO (g·d^−1^)		206.02 (103.13)		189.46 (79.35)		236.24 (131.21)	*p* < 0.001
Dietary fiber (g·d^−1^)		24.54 (12.45)		22.76 (10.68)		27.80 (14.64	*p* < 0.001
PRO (% energy)		21.83 (4.78)		21.83 (4.92)		21.84 (4.53)	*p* > 0.05
PRO (g·d^−1^)		106.62 (49.07)		59.48 (39.92)		126.94 (57.18)	*p* < 0.001
Fat (% energy)		35.51 (7.40)		34.72 (7.37)		36.96 (7.25)	*p* = 0.002
Fat (g·d^−1^)		75.86 (34.42)		67.54 (30.21)		91.05 (36.48)	*p* < 0.001
SFA (g·d^−1^)		22.24 (11.09)		19.88 (9.95)		26.54 (11.76)	*p* < 0.001
MUFA (g·d^−1^)		29.52 (14.54)		25.97 (12.78)		35.98 (15.36)	*p* < 0.001
PUFA (g·d^−1^)		17.23 (8.01)		15.53 (6.99)		20.34 (8.81)	*p* < 0.001
CHOL (mg·d^−1^)		356.87 (235.01)		309.74 (202.65)		442.84 (264.51)	*p* < 0.001
Alcohol (% energy)		2.18 (3.40)		2.10 (3.00)		2.33 (4.01)	*p* > 0.05
Alcohol (g·d^−1^)		5.48 (8.24)		4.89 (6.87)		6.57 (10.22)	*p* > 0.05
Micronutrient Score		0.09 (0.02)		0.10 (0.03)		0.09 (0.02)	*p* < 0.001
Energy (kcals·kg^−1^·d^−1^)	446	26.53 (10.74)	287	26.14 (10.02)	159	27.24 (11.91)	*p* > 0.05
CHO (g·kg^−1^·d^−1^)		2.85 (1.43)		2.85 (1.29)		2.85 (1.66)	*p* > 0.05
PRO (g·kg^−1^·d^−1^)		1.47 (0.65)		1.44 (0.62)		1.53 (0.71)	*p* > 0.05
Fat (g·kg^−1^·d^−1^)		1.05 (0.47)		1.01 (0.48)		1.10 (0.46)	*p* = 0.059
Alcohol (g·kg^−1^·d^−1^)		0.08 (0.11)		0.07 (0.10)		0.08 (0.13)	*p* > 0.05

Note: CHO, carbohydrate; PRO, protein; SFA, saturated fatty acids; MUFA, monounsaturated fatty acids; PUFA, polyunsaturated fatty acids; CHOL, cholesterol; kcals, kilocalories; kg, kilogram; g, gram; %, percentage of total energy.

**Table 3:** Summary of multiple linear regression formulae, with exercise habits and clinical outcomes as the dependent variables.

Dependent Variable	Formula
Total exercise sessions·week^−1^	7.59 + 1.10 (goal: CVE) − 1.05 (goal: OW)
Additional aerobic exercise sessions·week^−1^ other than CrossFit^®^	1.53 + 0.63 (goal: CVE) − 0.43 (goal: OW)
Additional strength exercise sessions·week^−1^ other than CrossFit^®^	1.73 + 0.39 (goal: CVE) − 0.81 (goal: OW)
Resting heart rate (bpm)	51.21 − 4.78 (sex) + 0.40 (BMI) − 0.52 (EXS)
Systolic blood pressure (mmHg)	99.15 + 6.99 (sex) + 0.47 (BMI)
Diastolic blood pressure (mmHg)	59.39 + 2.75 (sex) + 0.39 (BMI)

Note: BMI, body mass index (kg·m^2^); EXS, exercise frequency (sessions·week^−1^); (sex), sex is coded as 0 = female, 1 = male; CVE, fitness goal of cardiovascular endurance, coded as 0 = no, 1 = yes; OW, fitness goal of overall wellbeing, coded as 0 = no, 1 = yes.

**Table 4:** Relationship between sex, weight status, age, exercise frequency, nutritional goals, and fitness goals.

	Males vs. Females	BMI	Age	EXS
Lose fat mass	0.426 (0.277, 0.657)	1.126 (1.069, 1.187)	ns	ns
Weight loss	0.228 (0.125, 0.416)	1.285 (1.200, 1.377)	ns	ns
Weight maintenance	ns	0.943 (0.891, 0.999)	ns	0.916 (0.843, 0.944)
Support performance	ns	0.943 (0.899, 0.988)	ns	ns
Gain muscle mass	1.935 (1.275, 2.935)	0.929 (0.884, 0.976)	0.980 (0.963, 0.997)	ns
Weight gain	17.177 (4.623, 63.815)	0.752 (0.606, 0.933)	0.921 (0.869, 0.976)	ns
Cardiovascular Endurance	1.855 (1.141, 2.879)	ns	0.976 (0.958, 0.994)	1.147 (1.055, 1.246)
Flexibility	2.485 (1.664, 3.710)	ns	ns	1.079 (1.006, 1.157)
Strength	ns	ns	0.973 (0.953, 0.993)	ns
Overall well-being	ns	ns	1.032 (1.009, 1.057)	0.893 (0.832, 0.968)

Note: Data reported as odds ratio (95% CI). BMI, body mass index (kg·m^2^); EXS, exercise frequency (sessions·week^−1^); (sex) sex is coded as 0 = female, 1 = male; ns, not significant.

**Table 5:** Relationship between sex, weight status, age, exercise frequency, and clinical outcomes.

	Males vs. Females	BMI	Age	EXS
Diagnosis of mental disease	0.366 (0.203, 0.659)	1.067 (1.012, 1.124)	ns	ns
Diagnosis of cardiovascular disease	ns	1.156 (1.050, 1.273)	1.125 (1.071, 1.182)	ns
Diagnosis of metabolic disease	ns	1.181 (1.085, 1.284)	ns	ns
Diagnosis of skeletomuscular disease	ns	0.680 (0.514, 0.901)	ns	ns
Taking prescription medications (including birth control)	0.246 (0.153, 0.396)	ns	1.026 (1.008, 1.045)	ns
Change in diagnosis or medication after participating in CrossFit^®^	ns	1.103 (1.038, 1.172)	1.037 (1.010, 1.065)	ns
Change in disease symptoms after participating in CrossFit^®^	0.552 (0.332, 0.918)	1.077 (1.024, 1.133)	1.021 (1.001, 1.042)	ns

Note: Data reported as odds ratio (95% CI). BMI, body mass index (kg·m^2^); EXS, exercise frequency (sessions·week^−1^); (sex) sex is coded as 0 = female, 1 = male; ns, not significant.

## Data Availability

The datasets generated during and/or analyzed during the current work are not publicly available but are available from the corresponding author on reasonable request.

## Supplementary Materials

The following are available online at https://www.mdpi.com/article/10.3390/sports10030038/s1. File S1: Intake Survey, Table S1: Summary of multiple linear regression formulas with dietary intake as the dependent variables, Table S2: Correlations between dietary intake, exercise habits, resting heart rate, blood pressure, sex, BMI, exercise frequency, and nutritional and fitness goals, Table S3: Results of multiple linear regression analysis with absolute energy, and carbohydrate, fat, and alcohol percentages of total energy as the dependent variables, Table S4: Results of multiple linear regression analysis with absolute macronutrient and micronutrient score variables as the dependent variables, Table S5: Results of weighted multiple linear regression analysis with relative macronutrient variables as the dependent variables, Table S6: Results of multiple linear regression analysis with dietary fiber, SFA, MUFA, PUFA, and CHOL as the dependent variables, Table S7: Vitamin and mineral intake used to calculate micronutrient score, Table S8: Results of multiple linear regression analysis with total exercise sessions, and additional aerobic and strength exercise sessions as the dependent variables, Table S9: Results of multiple linear regression analysis with heart rate and blood pressure as the dependent variables.
