# Supplementary material for: Dietary Intake of Adults Who Participate in CrossFit® Exercise Regimens
Source: Sports (Basel). 2022 Mar 5;10(3):38. doi: 10.3390/sports10030038 (PMC8954808; doi:10.3390/sports10030038)
Supplement: Supplementary file 1 [file sports-10-00038-s001.zip › sports-1488048-supplementary.pdf]

Supplementary Material

File S1: Intake survey.

<u>Informed consent</u>

- Q1: UNIVERSITY OF GEORGIA - CONSENT FORM
    - o I agree to the above informed consent and begin the survey
    - o I do not agree to the above informed consent

<u>Inclusion criteria</u>

- Q2: Are you currently 19 or more years of age?
    - o Yes
    - o No
- Q3: Do you currently and have been participating in a regular CrossFit® exercise regimen for at least 6 months?
    - o Yes
    - o No

<u>Demographics</u>

- Q4: What is your e-mail address?
- Q5: What is your race?
    - o American Indian or Alaska Native
    - o Asian
    - o Black or African American
    - o Native Hawaiian or other Pacific Islander
    - o Hispanic
    - o Latino or Spanish origin
    - o White
    - o I prefer not to answer
- Q6: What is your height in inches?
- Q7: What is your weight in pounds?
- Q8: What is your body composition?
    - o Body composition is the proportion of fat and non-fat mass in your body. It is represented as a percentage of fat mass. If measuring your body composition at home, please list a measurement that was taken in the morning after you voided your bladder. If you do not know, please leave blank.
- Q9: What is your waist circumference in inches?
    - o To measure your waist circumference: stand and place a tape measure around your middle, just above your hipbones. Make sure tape is horizontal around the waist. Keep the tape snug around the waist, but not compressing the skin. If you do not know your waist circumference, please leave blank
- Q10: What is your hip circumference in inches?
    - o To measure your hip circumference: stand and place a tape measure around the largest part of your hips. Make sure tape is horizontal around the waist. Keep the tape snug around the hips, but not compressing the skin. If you do not know your hip circumference, please leave blank

Nutritional goals

- Q11: What is your current nutritional goal? Check all that apply.
    - o Weight loss
    - o Weight maintenance
    - o Weight gain
    - o Gain muscle mass
    - o Lose fat mass
    - o Support performance
    - o Other ________
- Q12: How long has this been your nutritional goal?

Exercise habits

- Q13: How many years have you been participating in CrossFit®?
- Q14: What is your current fitness goal? Check all that apply.
    - o Cardiovascular endurance
    - o Strength
    - o Overall well-being
    - o Flexibility
    - o Other:_______
- Q15: On average, how many days a week do you participate in:
    - o CrossFit® class (~1 hr in duration)?
    - o Additional strength specific training sessions (e.g., powerlifting or Olympic lifting) outside of a traditional CrossFit® class?
    - o Additional cardiovascular specific training sessions (e.g., running, cycling, etc) outside of a traditional CrossFit® class?
    - o Additional flexibility specific training sessions (e.g., yoga) outside of a traditional CrossFit® class?

Clinical outcomes

- Q16: What is your resting heart rate?
- Q17: What is your resting blood pressure?
- Q18: Have you been diagnosed with any of the following? Check all that apply.
    - o Hypertension
    - o Dyslipidemia
    - o Atherosclerosis
    - o Heart disease
    - o Heart attack or failure
    - o Stroke
    - o Low bone density
    - o Pre-diabetes
    - o Type-2 diabetes
    - o Cirrhosis of the liver
    - o Depression
    - o Anxiety disorder
    - o Attention deficit disorder (ADD)
    - o Attention deficit hyperactivity disorder (ADHD)

    - Post-traumatic stress disorder (PTSD)
- Q19: Please list all prescription medications and reason for taking.
- Q20: Since you have been participating in CrossFit®, has your physician change your diagnosis or medication for any previous diseases or disorders?
    - Yes
    - No
- Q21: Since you have been participating in CrossFit®, have you experienced a change in any symptoms associated with any disease or disorder?
    - Yes
    - No

Supplemental Table S1. Summary of multiple linear regression formulas with dietary intake as the dependent variables**.**

Dependent Variable	Formula
kcals·d^{-1}	2261.87 + 540.70 (sex) – 14.95 (age) + 411.19 (goal: WG)
kcals·kg^{-1}·d^{-1} (weighted)	46.21 – 0.64 (BMI) – 0.13 (age) + 0.34 (EXS) – 1.98 (goal: LFM) + 6.50 (goal: WG) – 2.56 (goal: WL)
CHO % energy	46.49 – 2.56 (sex) – 0.15 (age) + 0.32 (EXS) + 4.47 (goal: WG)
CHO g·d^{-1}	242.92 + 45.33 (sex) – 2.19 (age) + 3.98 (EXS) – 22.54 (goal: WL) + 66.98 (goal: WG)
CHO g·kg^{-1}·d^{-1} (weighted)	4.61 – 0.05 (BMI) – 0.02 (age) + 0.05 (EXS) – 0.41 (goal: WL) + 1.18 (goal: WG)
Fiber g·d^{-1}	24.76 + 5.23 (sex) – 0.21 (age) + 0.63 (EXS) – 3.23 (goal: LFM) + 3.09 (goal: SP)
PRO g·d^{-1}	109.34 + 34.04 (sex) – 0.77 (age) + 1.82 (EXS)
PRO g·kg^{-1}·d^{-1} (weighted)	4.61 + 0.20 (sex) – 0.05 (BMI) – 0.01 (age) + 0.02 (EXS)
Fat % energy	30.67 – 2.11 (sex) – 0.12 (age) – 3.52 (goal: WG)
Fat g·d^{-1} (weighted)	75.31 + 23.74 (sex) – 0.34 (age) + 6.26 (goal: SP)
Fat g·kg^{-1}·d^{-1} (weighted)	1.93 + 0.14 (sex) – 0.03 (BMI) – 0.004 (age)
SFA g·d^{-1} (weighted)	23.61 + 6.57 (sex) – 0.10 (age)
MUFA g·d^{-1} (weighted)	27.42 + 10.17 (sex) – 0.10 (age) + 2.73 (goal: SP)
PUFA g·d^{-1}	16.82 + 4.89 (sex) – 0.10 (age) + 0.41 (EXS) – 1.49 (goal: LFM)
CHOL mg·d^{-1} (weighted)	273.24 + 135.05 (sex) + 51.05 (goal: SP)
ALC % energy (weighted)	1.31 + 0.05 (age) – 0.11 (EXS)
ALC g·d^{-1} (weighted)	3.68 + 0.11 (age) – 0.31 (EXS)
ALC g·kg^{-1}·d^{-1} (weighted)	10.06 + 0.001 (age) – 0.004 (EXS)
Micronutrient Score	0.101 – 0.01 (sex) – 0.001 (BMI) + 0.001 (EXS) + 0.01 (goal: WL)

Note: Unstandardized Beta reported with (Std. Error). Weighted multiple linear regression is adjusted by amount of prediction error associated with the dependent variable to control for heteroscedasticity found in the unweighted model. CHO, carbohydrate; PRO, protein; ALC, alcohol; BMI, body mass index (kg·m^2); EXS, exercise frequency (sessions·wk^{-1}); Sex, sex is coded as 0 = female, 1 = male; LFM, nutritional goal of lose fat mass, coded as 0 = no, 1 = yes; WL, nutritional goal of weight loss, coded as 0 = no, 1 = yes; SP, nutritional goal of support performance, coded as 0 = no, 1 = yes; WG, nutritional goal of weight gain, coded as 0 = no, 1 = yes; g, gram; kg, kilogram; mg, milligram; d, day.

Supplemental Table S2: Correlations between dietary intake, exercise habits, resting heart rate, blood pressure, sex, BMI, age, exercise frequency, and nutritional and fitness goals.

	Sex	BMI	Age	EXS	LFM	WL	SP	WM	GMM	WG	CVE	OW	S
Energy (kcals·d^{-1})	0.316*	0.031	-0.180*	0.114*	-0.135*	-0.145*	0.081*	-0.044	0.028	0.185*	N/A	N/A	N/A
Energy (kcals·kg^{-1}·d^{-1}, weighted)	0.031	-0.315*	-0.128*	0.106*	-0.172*	-0.232*	0.095*	0.009	0.049	0.160*	N/A	N/A	N/A
CHO (% energy)	-0.136*	0.016	-0.220*	0.105*	-0.011	-0.033	-0.055	-0.027	0.025	0.092*	N/A	N/A	N/A
CHO (g·d^{-1})	0.219*	0.022	-0.233*	0.135*	-0.130*	-0.141*	0.047	-0.061	0.022	0.205*	N/A	N/A	N/A
CHO (g·kg^{-1}·d^{-1}, weighted)	0.026	-0.326*	-0.196*	0.158*	-0.190*	-0.292*	0.116*	0.002	0.034	0.207*	N/A	N/A	N/A
PRO (% energy)	0.003	-0.018	0.013	0.020	0.050	0.073	0.024	-0.055	-0.027	-0.065	N/A	N/A	N/A
PRO (g·d^{-1})	0.311*	0.042	-0.139*	0.130*	-0.081*	-0.096*	0.081*	-0.055	0.030	0.139*	N/A	N/A	N/A
PRO (g·kg^{-1}·d^{-1})	0.071	-0.254*	-0.107*	0.111*	-0.115*	-0.173*	0.126*	-0.004	0.017	0.095*	N/A	N/A	N/A
Fat (% energy)	0.144*	-0.016	0.206*	-0.063	-0.038	0.0001	0.067	0.035	-0.034	-0.088*	N/A	N/A	N/A
Fat (g·d^{-1}, weighted)	0.305*	0.029	-0.105*	0.071	-0.112*	-0.105*	0.092*	-0.017	0.012	0.117*	N/A	N/A	N/A
Fat (g·kg^{-1}·d^{-1}, weighted)	0.073	-0.372*	-0.054	0.098*	-0.159*	-0.262*	0.139*	0.015	0.058	0.114*	N/A	N/A	N/A
ALC (% energy, weighted)	-0.171*	-0.086*	0.331*	-0.369*	0.264*	0.186*	-0.159*	0.192*	0.144*	0.047	N/A	N/A	N/A
ALC (g·d^{-1}, weighted)	0.124*	-0.011	0.197*	-0.195*	0.065	0.001	-0.09	0.089*	0.029	0.022	N/A	N/A	N/A
ALC (g·kg^{-1}·d^{-1}, weighted)	0.042	-0.090*	0.183*	-0.201*	0.072	-0.028	0.012	0.096*	0.057	0.012	N/A	N/A	N/A
Dietary fiber (g·d^{-1})	0.198*	-0.052	-0.168*	0.176*	-0.159*	-0.129*	0.140*	-0.070	0.039	0.107*	N/A	N/A	N/A
SFA (g·d^{-1}, weighted)	0.265*	0.026	-0.088*	-0.007	-0.089*	-0.115*	0.090*	0.005	0.008	0.107*	N/A	N/A	N/A
MUFA (g·d^{-1}, weighted)	0.318*	0.007	-0.062	0.078*	-0.130*	-0.100*	0.094*	-0.027	0.015	0.095*	N/A	N/A	N/A
PUFA (g·d^{-1})	0.290*	0.037	-0.107*	0.169*	-0.131*	-0.105*	0.080*	-0.043	0.025	0.115*	N/A	N/A	N/A

CHOL (mg·d^{-1}, weighted)	0.249*	0.008	-0.015	-0.018	-0.033	-0.074†	0.104*	-0.002	-0.035	0.099*	N/A	N/A	N/A
Micronutrient Score	-0.189*	-0.131*	-0.025	0.110*	0.043	0.095*	0.079*	-0.088*	0.0001	-0.015	N/A	N/A	N/A
Total exercise sessions	0.042	0.011	-0.068	N/A	N/A	N/A	N/A	N/A	N/A	N/A	0.168*	-0.138*	0.083*
CrossFit® sessions·wk^{-1}	0.066	0.054	-0.020	N/A	N/A	N/A	N/A	N/A	N/A	N/A	0.031	0.060	-0.009
Additional aerobic sessions·wk^{-1}	-0.012	-0.026	-0.020	N/A	N/A	N/A	N/A	N/A	N/A	N/A	0.171*	-0.093*	0.038
Additional strength sessions·wk^{-1}	0.035	0.002	-0.086*	N/A	N/A	N/A	N/A	N/A	N/A	N/A	0.101*	-0.202*	0.118*
Resting heart rate (bpm)	-0.221*	0.137*	-0.016	-0.171*	N/A	N/A	N/A	N/A	N/A	N/A	N/A	N/A	N/A
Systolic blood pressure (mmHg)	0.365*	0.272*	0.065	-0.022	N/A	N/A	N/A	N/A	N/A	N/A	N/A	N/A	N/A
Diastolic blood pressure (mmHg)	0.216*	0.252*	-0.019	0.050	N/A	N/A	N/A	N/A	N/A	N/A	N/A	N/A	N/A

Note: BMI, body mass index (kg·m^2); EXS, exercise frequency (sessions·wk^{-1}); Sex, sex is coded as 0 = female, 1 = male; Nutritional goals coded as 0 = no, 1 = yes; LFM, nutritional goal of lose fat mas, coded as 0 = no, 1 = yes; WL, nutritional goal of weight loss, coded as 0 = no, 1 = yes; SP, nutritional goal of support performance, coded as 0 = no, 1 = yes; WM, nutritional goal of weight maintenance, coded as 0 = no, 1 = yes; GMM, nutritional goal of gain muscle mass, coded as 0 = no, 1 = yes; WG, nutritional goal of weight gain, coded as 0 = no, 1 = yes; CVE, fitness goal of cardiovascular endurance, coded as 0 = no, 1 = yes; OW, fitness goal of overall wellbeing, coded as 0 = no, 1 = yes; S, fitness goal of strength, coded as 0 = no, 1 = yes; CHO, carbohydrate; PRO, protein; ALC, alcohol; SFA, saturated fatty acids; MUFA, monounsaturated fatty acids; PUFA, polyunsaturated fatty acids; CHOL, cholesterol; kcals, kilocalories; N/A, not applicable; kg, kilogram; g, gram; % energy, percent of total energy·d^{-1}; bpm, beats·min^{-1}; mmHg, millimeters of mercury. Weighted multiple linear regression is adjusted by amount of prediction error associated with the dependent variable to control for heteroscedasticity found in the unweighted model. *$P \leq 0.05$ (1-tailed); † $P = 0.060$.

Supplemental Table S3 Results of multiple linear regression analysis with absolute energy, and carbohydrate, fat, and alcohol percent of total energy as the dependent variables

Step	Dependent variables											
	Energy (kcals·d^{-1})			CHO (% energy)			Fat (% energy)			ALC (% energy, weighted)		
	IV	B	*P*	IV	B	*P*	IV	B	*P*	IV	B	*P*
1	Sex	521.19 (74.60)	<0.001	Age	-0.18 (0.04)	<0.001	Age	0.14 (0.03)	<0.001	EXS	-0.15 (0.02)	<0.001
2	Sex	574.85 (73.26)	<0.001	Age	-0.17 (0.04)	<0.001	Age	0.12 (0.03)	<0.001	EXS	-0.11 (0.02)	<0.001
	Age	-15.91 (3.08)	<0.001	Sex	-2.09 (0.91)	0.023	Sex	1.81 (0.73)	0.013	Age	0.05 (0.01)	<0.001
3	Sex	540.70 (74.42)	<0.001	Age	-0.16 (0.04)	<0.001	Age	0.12 (0.03)	<0.001			
	Age	-14.95 (3.10)	<0.001	Sex	-2.18 (0.91)	0.017	Sex	2.11 (0.74)	0.005			
	WG	411.19 (179.47)	0.022	EXS	0.32 (0.15)	0.039	WG	-3.52 (1.78)	0.049			
4				Age	-0.15 (0.04)	<0.001						
				Sex	-2.56 (0.93)	0.006						
				EXS	0.32 (0.15)	0.038						
				WG	4.47 (2.23)	0.046						
Total R^2		0.161			0.077			0.064			0.165	

Note: Unstandardized Beta reported with (Std. Error). Weighted multiple linear regression is adjusted by amount of prediction error associated with the dependent variable to control for heteroscedasticity found in the unweighted model. EXS, exercise frequency (sessions·wk^{-1}); Sex, coded as 0 = female, 1 = male; WG, nutritional goal of weight gain, coded as 0 = no, 1 = yes; kcals, kilocalories; CHO, carbohydrate; ALC, alcohol; % energy, percent of total energy·d^{-1}.

Supplemental Table S4 Results of multiple linear regression analysis with absolute macronutrient and micronutrient score variables as the dependent variables

Step	Dependent variables														
	CHO (g·d^{-1})			PRO (g·d^{-1})			Fat (g·d^{-1}, weighted)			ALC (g·d^{-1}, weighted)			Micronutrient Score		
	IV	B	*P*	IV	B	*P*	IV	B	*P*	IV	B	*P*	IV	B	*P*
1	Age	-2.11 (0.42)	<0.001	Sex	31.89 (4.65)	<0.001	Sex	22.76 (3.35)	<0.001	Age	0.13 (0.03)	<0.001	Sex	-0.01 (0.002)	<0.001
2	Age	-2.44 (0.41)	<0.001	Sex	34.61 (4.61)	<0.001	Sex	23.75 (3.33)	<0.001	Age	0.11 (0.03)	<0.001	Sex	-0.10 (0.002)	<0.001
	Sex	55.49 (9.75)	<0.001	Age	-0.81 (0.19)	<0.001	Age	-0.35 (0.11)	0.003	EXS	-0.31 (0.09)	<0.001	EXS	0.001 (0.0001)	0.011
3	Age	-2.28 (0.41)	<0.001	Sex	34.04 (4.60)	<0.001	Sex	23.74 (3.37)	<0.001				Sex	-0.01 (0.002)	<0.001
	Sex	49.58 (9.87)	<0.001	Age	-0.77 (0.19)	<0.001	Age	-0.34 (0.11)	0.003				EXS	0.001 (0.0001)	0.011
	WG	71.17 (23.80)	0.003	EXS	1.82 (0.78)	0.020	SP	6.26 (3.15)	0.048				BMI	-0.001 (0.001)	0.039
4	Age	-2.20 (0.41)	<0.001										Sex	-0.01 (0.003)	0.004
	Sex	48.36 (9.83)	<0.001										EXS	0.001 (0.0001)	0.011
	WG	71.10 (23.67)	0.003										BMI	-0.001 (0.0001)	0.003
	EXS	3.93 (1.63)	0.016										WL	0.01 (0.003)	0.013
5	Age	-2.19 (0.41)	<0.001												
	Sex	45.33 (9.90)	<0.001												
	WG	66.98 (23.66)	0.005												
	EXS	3.98 (1.63)	0.015												
	WL	-22.54 (10.89)	0.039												
Total R^2		0.156			0.141			0.119			0.064			0.072	

Note: Unstandardized Beta reported with (Std. Error). Weighted multiple linear regression is adjusted by amount of prediction error associated with the dependent variable to control for heteroscedasticity found in the unweighted model. BMI, body mass index (kg·m^2); EXS, exercise frequency (sessions·wk^{-1}); Sex, sex is coded as 0 = female, 1 = male; WG, nutritional goal of weight gain, coded as 0 = no, 1 = yes; WL, nutritional goal of weight loss, coded as 0 = no, 1 = yes; SP, nutritional goal of support performance, coded as 0 = no, 1 = yes; kcals, kilocalories; kg, kilogram; g, gram; CHO, carbohydrate; PRO, protein; ALC, alcohol; %, percent of total energy·d^{-1}.

Supplemental Table S5 Results of weighted multiple linear regression analysis with relative macronutrient variables as the dependent variables

Step	Dependent variables														
	Energy (kcals·kg^{-1}·d^{-1}, (weighted)			CHO (g·kg^{-1}·d^{-1}, weighted)			PRO (g·kg^{-1}·d^{-1}, weighted)			Fat (g·kg^{-1}·d^{-1}, weighted)			ALC (g·kg^{-1}·d^{-1}, weighted)		
	IV	B	*P*	IV	B	*P*	IV	B	*P*	IV	B	*P*	IV	B	*P*
1	BMI	-0.75 (0.11)	<0.001	BMI	-0.07 (0.01)	<0.001	BMI	-0.04 (0.01)	<0.001	BMI	-0.03 (0.003)	<0.001	EXS	-0.01 (0.001)	<0.001
2	BMI	-0.79 (0.11)	<0.001	BMI	-0.07 (0.01)	<0.001	BMI	-0.04 (0.01)	<0.001	BMI	-0.03 (0.003)	<0.001	EXS	-0.004 (0.001)	<0.001
	Age	-0.14 (0.04)	<0.001	Age	-0.03 (0.01)	<0.001	Age	-0.01 (0.003)	0.006	Sex	0.13 (0.04)	0.002	Age	0.001 (0.0001)	0.002
3	BMI	-0.74 (0.11)	<0.001	BMI	-0.06 (0.01)	<0.001	BMI	-0.04 (0.01)	<0.001	BMI	-0.03 (0.003)	<0.001			
	Age	-0.15 (0.04)	<0.001	Age	-0.03 (0.01)	<0.001	Age	-0.01 (0.003)	<0.001	Sex	0.14 (0.04)	<0.001			
	LFM	-2.67 (0.91)	0.003	WG	1.28 (0.35)	<0.001	Sex	0.21 (0.06)	0.001	Age	-0.004 (0.002)	0.011			
4	BMI	-0.73 (0.11)	<0.001	BMI	-0.05 (0.01)	0.001	BMI	-0.05 (0.01)	<0.001						
	Age	-0.13 (0.04)	<0.001	Age	-0.03 (0.01)	0.001	Age	-0.01 (0.003)	0.021						
	LFM	-2.29 (0.92)	0.013	WG	1.19 (0.35)	0.001	Sex	0.20 (0.06)	0.002						
	WG	6.89 (2.82)	0.015	WL	-0.42 (0.13)	0.002	EXS	0.02 (0.01)	0.020						
5	BMI	-0.64 (0.11)	<0.001	BMI	-0.05 (0.01)	<0.001									
	Age	-0.13 (0.04)	<0.001	Age	-0.02 (0.01)	<0.001									
	LFM	-2.02 (0.92)	0.029	WG	1.18 (0.35)	<0.001									
	WG	6.56 (2.81)	0.020	WL	-0.41 (0.13)	0.002									
	WL	-2.53 (1.14)	0.027	EXS	0.05 (0.02)	0.018									
6	BMI	-0.64 (0.11)	<0.001												
	Age	-0.13 (0.04)	<0.001												
	LFM	-1.98 (0.92)	0.031												
	WG	6.50 (2.80)	0.020												
	WL	-2.56 (1.13)	0.024												
	EXS	0.34 (0.15)	0.030												
Total R^2		0.173			0.207			0.112			0.169			0.062	

Note: Unstandardized Beta reported with (Std. Error). Weighted multiple linear regression is adjusted by amount of prediction error associated with the dependent variable to control for heteroscedasticity found in the unweighted model. BMI, body mass index ($kg\cdot m^2$); EXS, exercise frequency ($sessions\cdot wk^{-1}$); Sex, sex is coded as 0 = female, 1 = male; LFM, nutritional goal of lose fat mass, coded as 0 = no, 1 = yes; WG, nutritional goal of weight gain, coded as 0 = no, 1 = yes; WL, nutritional goal of weight loss, coded as 0 = no, 1 = yes; kcals, kilocalories; kg, kilogram; g, gram; CHO, carbohydrate; PRO, protein; ALC, alcohol; %, percent of total $energy\cdot d^{-1}$.

Supplemental Table S6 Results of multiple linear regression analysis with dietary fiber, SFA, MUFA, PUFA, and CHOL as the dependent variables

Step	Dependent variables														
	Dietary Fiber ($g\cdot d^{-1}$)			SFA ($g\cdot d^{-1}$, weighted)			MUFA ($g\cdot d^{-1}$, weighted)			PUFA ($g\cdot d^{-1}$)			CHOL ($mg\cdot d^{-1}$, weighted)		
	IV	B	*P*	IV	B	*P*	IV	B	*P*	IV	B	*P*	IV	B	*P*
1	Sex	5.18 (1.22)	<0.001	Sex	6.24 (1.08)	<0.001	Sex	9.88 (1.41)	<0.001	Sex	4.87 (0.77)	<0.001	Sex	135.38 (25.04)	<0.001
2	Sex	5.92 (1.21)	<0.001	Sex	6.57 (1.08)	<0.001	Sex	9.89 (1.40)	<0.001	Sex	4.65 (0.76)	<0.001	Sex	135.05 (24.93)	<0.001
	Age	-0.22 (0.05)	<0.001	Age	-0.10 (0.04)	0.009	SP	2.74 (1.33)	0.039	EXS	0.45 (0.13)	<0.001	SP	51.05 (22.91)	0.026
3	Sex	5.71 (1.20)	<0.001				Sex	10.17 (1.40)	<0.001	Sex	5.10 (0.76)	<0.001			
	Age	-0.21 (0.05)	<0.001				SP	2.73 (1.32)	0.039	EXS	0.42 (0.13)	0.001			
	EXS	0.69 (0.20)	<0.001				Age	-0.10 (0.05)	0.039	Age	-0.10 (0.03)	0.002			
4	Sex	5.23 (1.20)	<0.001							Sex	4.89 (0.76)	<0.001			
	Age	-0.21 (0.05)	<0.001							EXS	0.41 (0.13)	0.001			
	EXS	0.66 (0.20)	0.001							Age	-0.10 (0.03)	0.001			
	LFM	-3.43 (1.15)	0.003							LFM	-1.49 (0.73)	0.042			
5	Sex	5.23 (1.19)	<0.001												
	Age	-0.21 (0.05)	<0.001												

EXS	0.63 (0.20)	0.002								
LFM	-3.23 (1.14)	0.005								
SP	3.09 (1.24)	0.013								
Total R^2	0.132		0.084		0.118		0.136		0.073	

Note: Unstandardized Beta reported with (Std. Error). Weighted multiple linear regression is adjusted by amount of prediction error associated with the dependent variable to control for heteroscedasticity found in the unweighted model. BMI, body mass index ($kg \cdot m^2$); EXS, exercise frequency ($sessions \cdot wk^{-1}$); Sex, sex is coded as 0 = female, 1 = male; LFM, nutritional goal of lose fat mass, coded as 0 = no, 1 = yes; SP, nutritional goal of support performance, coded as 0 = no, 1 = yes; SFA, saturated fatty acids; MUFA, monounsaturated fatty acids; PUFA, polyunsaturated fatty acids; CHOL, cholesterol; g, gram; mg, milligram.

Supplemental Table S7: Vitamin and mineral intake used to calculate micronutrient score.

Micronutrient	All (*n* = 449)	Female (*n* = 290)	Male (*n* = 159)	ANOVA
Vitamin A ($\mu g \cdot d^{-1}$)	543.63 (394.12)	474.20 (278.52)	670.27 (522.84)	$P < 0.001$
Vitamin B_1 ($mg \cdot d^{-1}$)	1.88 (0.88)	1.76 (0.77)	2.09 (1.02)	$P < 0.001$
Vitamin B_2 ($mg \cdot d^{-1}$)	3.01 (1.34)	2.80 (1.18)	3.39 (1.52)	$P < 0.001$
Vitamin B_3 ($mg \cdot d^{-1}$)	31.50 (17.08)	28.67 (15.97)	36.66 (17.86)	$P < 0.001$
Vitamin B_5 ($mg \cdot d^{-1}$)	8.16 (3.72)	7.32 (2.98)	9.69 (4.40)	$P < 0.001$
Vitamin B_6 ($mg \cdot d^{-1}$)	3.06 (1.88)	2.82 (1.83)	3.51 (1.90)	$P < 0.001$
Vitamin B_9 ($\mu g \cdot d^{-1}$)	503.85 (253.62)	462.85 (217.26)	578.63 (295.51)	$P < 0.001$
Vitamin B_{12} ($\mu g \cdot d^{-1}$)	6.13 (4.72)	5.32 (4.01)	7.61 (5.52)	$P < 0.001$
Vitamin C ($mg \cdot d^{-1}$)	143.20 (92.44)	135.14 (80.92)	157.89 (109.16)	$P = 0.012$
Vitamin D ($\mu g \cdot d^{-1}$)	5.99 (4.31)	5.37 (3.76)	7.14 (4.98)	$P < 0.001$
Vitamin E ($mg \cdot d^{-1}$)	13.71 (7.49)	12.64 (6.85)	15.68 (8.20)	$P < 0.001$
Vitamin K ($\mu g \cdot d^{-1}$)	433.19 (476.26)	426.56 (495.74)	445.28 (439.76)	$P > 0.05$
Choline ($mg \cdot d^{-1}$)	464.09 (213.89)	413.20 (174.89)	556.91 (245.66)	$P < 0.001$
Calcium ($mg \cdot d^{-1}$)	1256.08 (570.15)	1173.51 (477.74)	1406.69 (684.79)	$P < 0.001$
Copper ($mg \cdot d^{-1}$)	1.90 (0.86)	1.71 (0.65)	2.23 (1.08)	$P < 0.001$
Iron ($mg \cdot d^{-1}$)	16.20 (7.54)	14.85 (6.17)	18.67 (9.06)	$P < 0.001$
Magnesium ($mg \cdot d^{-1}$)	448.28 (185.72)	416.03 (155.72)	507.11 (219.22)	$P < 0.001$
Manganese ($mg \cdot d^{-1}$)	4.16 (2.06)	3.90 (1.77)	4.64 (2.44)	$P < 0.001$
Phosphorus ($mg \cdot d^{-1}$)	1692.47 (715.87)	1535.36 (565.82)	1979.02 (859.98)	$P < 0.001$
Potassium ($mg \cdot d^{-1}$)	3472.13 (1488.32)	3203.33 (1210.12)	3962.39 (1796.56)	$P < 0.001$
Selenium ($\mu g \cdot d^{-1}$)	120.19 (56.69)	107.80 (46.69)	142.78 (65.81)	$P < 0.001$
Sodium ($mg \cdot d^{-1}$)	3527.09 (1524.35)	3205.50 (1302.75)	4113.65 (1717.04)	$P < 0.001$
Zinc ($mg \cdot d^{-1}$)	13.21 (6.12)	11.61 (4.53)	16.12 (7.46)	$P < 0.001$

Note: µg, microgram; mg, milligram; Vitamin B_1, Thiamin; Vitamin B_2, Riboflavin, Vitamin B_3, Niacin; Vitamin B_5, Pantothenic Acid; Vitamin B_6, Pyridoxine; Vitamin B_9, Folate; Vitamin B_{12}, Cobalamin.

Supplemental Table S8 Results of multiple linear regression analysis with total exercise sessions, and additional aerobic and strength exercise sessions as the dependent variables

Step	Dependent variables								
	Total exercise sessions			Additional aerobic sessions			Additional strength sessions		
	IV	B	*P*	IV	B	*P*	IV	B	*P*
1	CVE	1.03 (0.29)	<0.001	CVE	0.61 (0.17)	<0.001	OW	-0.78 (0.18)	<0.001
2	CVE	1.10 (0.29)	<0.001	CVE	0.63 (0.17)	<0.001	OW	-0.81 (0.18)	<0.001
	OW	-1.05 (0.33)	0.001	OW	-0.43 (0.19)	0.024	CVE	0.39 (0.16)	0.013
Total R^2		0.051			0.041			0.054	

Note: Unstandardized Beta reported with (Std. Error). CVE, fitness goal of cardiovascular endurance, coded as 0 = no, 1 = yes; OW, fitness goal of overall wellbeing, coded as 0 = no, 1 = yes; IV, independent variable.

Supplemental Table S9 Results of multiple linear regression analysis with heart rate and blood pressure as the dependent variables

Step	Dependent variables								
	Resting Heart Rate (bpm)			SBP (mmHg)			DBP (mmHg)		
	IV	B	*P*	IV	B	*P*	IV	B	*P*
1	Sex	-4.12 (0.92)	<0.001	Sex	8.06 (1.29)	<0.001	BMI	0.47 (0.11)	<0.001
2	Sex	-4.88 (0.92)	<0.001	Sex	6.99 (1.31)	<0.001	BMI	0.39 (0.12)	<0.001
	BMI	0.40 (0.10)	<0.001	BMI	0.47 (0.15)	0.001	Sex	2.75 (1.04)	0.009
3	Sex	-4.78 (0.91)	<0.001						
	BMI	0.40 (0.10)	<0.001						
	EXS	-0.52 (0.15)	<0.001						
Total R^2		0.112			0.168			0.088	

Note: Unstandardized Beta reported with (Std. Error). BMI, body mass index ($kg \cdot m^2$); EXS, exercise frequency ($sessions \cdot wk^{-1}$); Sex, sex is coded as 0 = female, 1 = male; SBP, systolic blood pressure; DBP, diastolic blood pressure; bpm, $beats \cdot min^{-1}$; mmHg, millimeters of mercury.
